# Dapagliflozin for the treatment of heart failure with reduced ejection fraction in Brazil: a cost-effectiveness analysis

**DOI:** 10.1016/j.lana.2024.100968

**Published:** 2024-12-28

**Authors:** Marcio Coutinho Xavier Naves, Angelica Amorim Amato, Ivan Ricardo Zimmermann, Henry Maia Peixoto

**Affiliations:** a School of Health Sciences, University of Brasilia, Brasilia, Brazil; b Laboratory of Molecular Pharmacology, University of Brasilia, Brasilia, Brazil; c Department of Public Health, University of Brasilia, Brasilia, Brazil; d National Institute of Science and Technology for Health Technology Assessment, Porto Alegre, Brazil; e Center for Tropical Medicine, University of Brasilia, Brasilia, Brazil

**Keywords:** Heart failure, Dapagliflozin, Cost-effectiveness, QALY, Brazil

## Abstract

**Background:**

Heart failure, a complex clinical syndrome with high morbidity and mortality, has become a significant burden on public health. Recently, a new class of antidiabetic agents–the sodium-glucose cotransporter 2 (SGLT2) inhibitors–was associated with a significant reduction on mortality and hospitalization in HF with reduced ejection fraction (HFrEF) when added to standard pharmacological treatment. Considering the lack of data on its cost-effectiveness, the present study aims to estimate the incremental cost-effectiveness ratio of add-on dapagliflozin treatment for HFrEF from the Brazilian public healthcare system perspective.

**Methods:**

We built a Markov model to estimate the clinical outcomes and costs of 1,000 hypothetical subjects with established HFrEF in a lifetime horizon. The model inputs were based on the Dapagliflozin and Prevention of Adverse Outcomes in Heart Failure (DAPA-HF) trial and local data. The main outcome was the incremental cost-effectiveness ratio (ICER) per quality-adjusted life year (QALY) gained. Deterministic and probabilistic sensitivity analyses, as well as scenario analyses, were performed.

**Findings:**

The addition of dapagliflozin to standard care treatment in 1,000 HFrEF patients yielded an expected value of 366.99 additional QALYs at an incremental cost of US$ 1,517,878.49, resulting in an ICER of US$ 4,136.08 per QALY gained, being a cost-effective strategy considering the Brazilian official cost-effectiveness threshold (US$ 8,000/QALY). In probabilistic sensitivity analyses, 96.60% of the simulations were also cost-effective. In the scenario analyses, results were similar for individuals with and without diabetes.

**Interpretation:**

Dapagliflozin is likely to be cost-effective when added to standard HFrEF therapy in Brazil.

**Funding:**

This study was supported by the National Institute of Science and Technology for Health Technology Assessment (Instituto de Avaliação de Tecnologias em Saúde—IATS).

**Research in context**

**Evidence before this study**

Sodium-glucose cotransporter-2 (SGLT2) inhibitors, originally developed for the treatment of type 2 diabetes, have been demonstrated to enhance cardiovascular outcomes in several clinical trials. Previous economic studies have provided evidence that the SGLT2 inhibitor dapagliflozin is cost-effective when added to heart failure with reduced ejection fraction standard therapy, with the majority of these studies conducted in high-income countries. A systematic search was conducted in PubMed and Google Scholar to identify studies evaluating the cost-effectiveness of dapagliflozin in patients with heart failure. The search strategy included the terms (“heart failure” OR “HF”) AND (“cost” OR “cost-effectiveness” OR “economic” OR “cost utility”) AND (“QALY” OR “quality-adjusted life year”). Additional searches were performed using Google and by reviewing references in identified studies. The review was limited to economic studies published after January 1, 2020 and before June 1, 2023. A total of 10 economic studies and one systematic review were identified, with only one study originating from Latin America (Colombia). However, the restricted external validity of cost-effectiveness studies limits the direct applicability of their findings across diverse settings, thereby underscoring the necessity for research that considers local social, economic, and epidemiological factors. This highlights the importance of conducting studies in Latin America and countries with universal health systems, such as Brazil.

**Added value of this study**

A state-transition model was developed to estimate the clinical outcomes and costs of 1,000 hypothetical subjects with established heart failure and reduced ejection fraction in a lifetime horizon, considering the Brazilian public healthcare system perspective. We gathered local data on treatment costs, in-hospital mortality, and all-cause death. The ICER of US$ 4,136,08 per QALY gained estimated in our study, below the Brazilian cost-effectiveness threshold of US$ 8,000 per QALY gained, indicates that dapagliflozin is cost-effective as an add-on therapy for heart failure in Brazil.

**Implications of all the available evidence**

Our results are consistent with other economic studies and add to the expanding body of evidence that supports the cost-effectiveness of dapagliflozin in heart failure. These findings have significant implications for the field of heart failure management, a disease with a tremendous global burden and responsible for a great part of the total health expenditure on cardiovascular diseases. The results of this study may help decision-makers in countries with social, epidemiological, and economic characteristics similar to Brazil to address the challenges of managing the burden of heart failure with limited budgets. Furthermore, the proposed methodology can be readily adapted to reflect the specifics of the local context.

## Introduction

Heart Failure is a complex clinical syndrome characterized by the inability of the ventricle to fill with or eject blood, resulting from many structural or functional cardiac diseases of high prevalence.^1^ It affects more than 56 million people worldwide, and its incidence is comparable to the four most common causes of cancer combined.^2^^,^^3^ The prevalence has increased over time due to improved survival after diagnosis of heart failure, global population growth, and aging of the population.^4^ Accordingly, the economic burden of heart failure is expected to rise.^5^ It was estimated that the global overall cost of heart failure in 2012 was at US$ 108 billion, with repeated hospitalizations accounting for the majority of these costs and turning it into a major challenge for healthcare systems.^6^ Despite the scarcity of evidence and the heterogeneity between studies regarding the epidemiology of heart failure in Latin America and the Caribbean, a meta-analysis found high mortality and hospitalization rates for the region. The prevalence of heart failure was 1% and the 1-year fatality rate was as high as 24.5%.^7^ In Brazil heart failure is a leading cause of hospital admission, accounting for more than 4% of all hospitalizations among patients older than 65 years. Brazilian patients admitted with decompensated heart failure experience longer hospital stays and higher in-hospital mortality rates compared to patients in the United States.^8^ The total direct costs for the Brazilian public health system (SUS) related to heart failure hospital care in 2022 were almost at US$ 90 million.^9^

The current optimum treatment of patients with heart failure and reduced ejection fraction (HFrEF), which encompasses almost half of heart failure patients and is associated with higher mortality rates, includes several classes of pharmacological agents and medical devices. Renin-angiotensin system agents (preferably coupled with a neprilysin inhibitor for enhanced benefit), beta-blockers, and mineralocorticoid receptor antagonists (MRA) have all demonstrated favorable long-term effects on morbidity and mortality. More recently, a new class of antidiabetic agents—the sodium glucose cotransporter 2 (SGLT2) inhibitors—were found to have favorable cardiovascular outcomes in multiple trials. The Dapagliflozin and Prevention of Adverse-outcomes in Heart Failure Trial (DAPA-HF) demonstrated that dapagliflozin added to standard HFrEF therapy significantly reduced the risk of cardiovascular death and hospitalization for heart failure, besides improving symptoms and quality of life when compared to placebo, irrespective of glycemic status.^10^ The mechanisms underlying the cardioprotective effects of SGLT2 inhibitors remain incompletely understood. While their glucose-lowering and diuretic effects, driven by their primary action on the nephron, are significant, they cannot fully explain the rapid and substantial benefits observed in the DAPA-HF and other major outcome trials.^11^ Additional metabolic effects, such as enhanced cardiac fuel energetics and improved calcium handling, as well as hormonal effects like increased glucagon release, may also contribute to these outcomes and appear to play potential roles in the treatment of heart failure.^12^

Considering the increasing economic burden of heart failure, it would be of great interest to conduct health economic evaluations to assess the efficiency of SGLT2 inhibition. Through reconciling its additional acquisition costs with its projected healthcare benefits, especially the averting of heart failure hospitalizations, an economic evaluation would support policymakers, payers, and providers. Among the various types of economic evaluation in health, cost-effectiveness analysis stands out by comparing the costs and outcomes of interventions using natural units, such as years of life saved. Its variant, cost-utility analysis, employs quality-adjusted life years (QALYs) as the outcome measure. The term “cost-effectiveness” is frequently used in a more expansive manner to encompass studies that, in addition to QALYs, incorporate other outcomes, thereby rendering it a more comprehensive term. The primary metric employed in both cost-effectiveness and cost-utility analysis is the incremental cost-effectiveness ratio (ICER), which is obtained by dividing the difference in costs (incremental cost) by the difference in health outcomes (incremental health outcome) between two technologies under evaluation. This ratio represents the cost per additional unit of health benefit achieved.^13^

Economical evaluations of dapagliflozin in HFrEF were reported in several countries, with most of them indicating that dapagliflozin is cost-effective as an add-on therapy for HFrEF.^14^, ^15^, ^16^, ^17^, ^18^ Nevertheless, these results should be analyzed with caution outside their original target population since there are relevant differences in heart failure regarding epidemiological data and treatment costs across distinct countries. Like other countries in Latin America and the Caribbean, the Brazilian Public Health System struggles with a higher incidence of chronic noncommunicable diseases and an unfinished agenda of tropical and neglected diseases, both of which affect the incidence of heart failure.^19^ Notedly, rheumatic heart disease and Chagas cardiomyopathy are still remaining as common causes of heart failure in many countries in the region.^20^ Therefore, the objective of this study is to estimate the incremental cost-effectiveness ratio (ICER) of adding dapagliflozin to standard therapy compared to standard therapy alone in patients with chronic HFrEF in Brazil.

## Methods

### Study design and evaluated strategies

The cost-effectiveness analysis considers the Brazilian public healthcare system (SUS) perspective. Given the chronic and progressive nature of heart failure, our analysis adopted a lifetime analytic horizon. We built a state-transition model to simulate the disease progression of patients with HFrEF, comparing two treatment strategies: the first based on the standard HFrEF therapy (standard therapy) and the second based on dapagliflozin (10 mg daily) added to the standard HFrEF therapy (dapagliflozin therapy). The standard therapy for HFrEF management included optimized doses of renin-angiotensin system agents (losartan, enalapril, or sacubitril/valsartan), beta-blockers (carvedilol), spironolactone, digoxin, and furosemide. It is in accordance with the Brazilian HF Guidelines published by the Ministry of Health and the Brazilian Cardiology Society.^21^

The primary outcome was measured as the incremental cost-effectiveness ratio (ICER) of dapagliflozin as an add-on therapy among HFrEF patients, calculated as incremental cost divided by incremental QALYs. We established a cost-effectiveness threshold of US$ 8,000 per QALY gained, in accordance with the Brazilian Ministry of Health recommendation.^22^ Other outcomes of interest were years of life saved and the number of hospitalizations avoided. Years of life saved represent the additional years a patient in the add-on dapagliflozin group is expected to live, compared to a patient in the standard HFrEF therapy. It was calculated as the difference in life expectancy between the two groups. The number of hospitalizations avoided represents the reduction in hospital admissions attributable to dapagliflozin, calculated as the difference in hospitalizations between the standard therapy and dapagliflozin therapy. The CHEERS checklist was employed to ensure all reporting standards for economic evaluations were met, details are included in the Supplemental material.

### Population

Our hypothetical cohort included 1,000 patients with similar characteristics to the DAPA-HF trial. Thus, in the first cycle all the patients had chronic HFrEF and were 66 years old. DAPA-HF was a randomized, double-blind, placebo-controlled, and event-driven trial that enrolled patients older than 18 years with established and symptomatic HFrEF (New York Heart Failure functional class II to IV and a left ventricular ejection fraction of 40% or less) that were optimally treated with standard pharmacological therapy. Patients were then randomized to receive dapagliflozin 10 mg daily or placebo in addition to standard therapy, with a median follow-up of 18.2 months. Further details on the baseline characteristics of the subjects included in the DAPA-HF can be found elsewhere.^10^^,^^23^

This present health economic modeling study did not require ethical approval, as it exclusively utilized secondary public data identified from published studies and anonymized databases.

### Model description

**Fig. 1:**
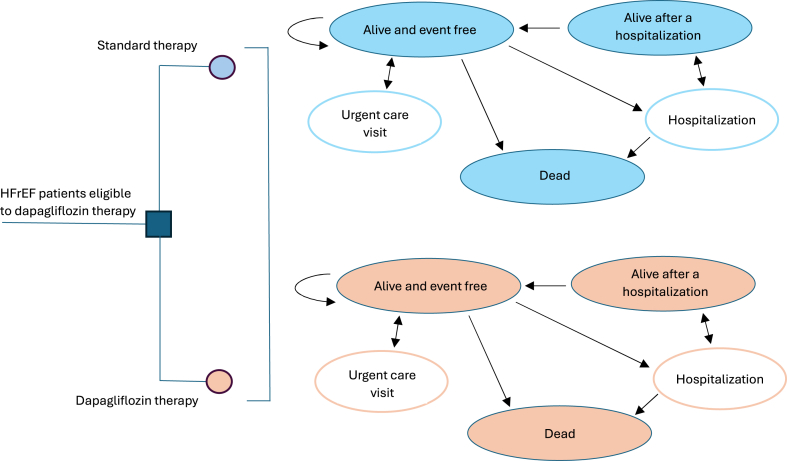
Model structure.

Our state-transition model used a Markov framework comprising three mutual health states: ‘stable disease’, ‘post-hospitalization due to heart failure’, and ‘death’. All the subjects start the model in the ‘stable disease’ state and then could remain in this state being exposed to the risk of experiencing a heart failure urgent care visit, a heart failure hospitalization, or dying from any cause (Fig. 1). A full definition of heart failure hospitalization and urgent care visit can be found at the supplementary methods.

In each cycle length of three months, the cohort accumulates quality-adjusted life years (QALYs) and costs based on the occurrence of these mutually exclusive health states and events. When hospitalized, the patients face the probability of dying (in-hospital mortality) or making the transition to the specific ‘post-hospitalization due to heart failure’ health state. In this state we assumed that there is an increased risk of readmission in the very next cycle, consistent with clinical data from heart failure patients.^24^^,^^25^ If not readmitted, the patients would subsequently return to the ‘stable disease’ state. Modelling was performed in TreeAge Pro Healthcare version 2024 R1 (TreeAge Software, Williamstown, Massachusetts).

### Clinical parameters

#### All-cause mortality

In the DAPA-HF trial, all-cause death occurred in a rate of 9.5 events per 100 patients/year in the control arm, during the 18.2-month study follow-up. Based on the declining exponential approximation of life expectancy (DEALE) principle,^26^ we compared this observed mortality rate with the aged-matched mortality rate of the Brazilian population, obtained from the 2022 Brazilian Population Census published by the Brazilian Institute of Geographic and Statistics.^27^ The yielded mortality rate ratio of 4.6 was assumed to be sustained after the trial period ended. We then applied this rate ratio in the Brazilian age-specific mortality data, thereby estimating the age-specific survival in the control arm during and beyond trial horizon. The annual rates were converted into quarterly transition probabilities, matching the model cycle length. This conversion process used the standard exponential relationship between rates and the corresponding probabilities.^28^^,^^29^ Complete mortality data stratified by age and diabetes diagnosis can be found in Supplementary Table S1. For the dapagliflozin arm, the age-specific mortality rates were obtained applying the hazard-ratio estimates from the DAPA-HF trial. Further detail on our model parameters are available in Table 1.

**Table 1:** Model parameters and range for sensitivity analysis.

Parameter	Base-case	Variation	Distribution	Source
**Standard therapy**				
Probability of urgent care visit	0.002^a^	0.001–0.003	Beta	^10^
Probability of HF hospitalization	0.024^b^	0.022–0.026	Beta	^10^
Probability of all-cause death	0.021^c^	0·019–0·023	Beta	^10^
Probability of death when hospitalized	0.09	0.08–0.10	Beta	^30^
Probability of readmission after a HF hospitalization	0.246	0.209–0.287	Beta	^25^
**Effectiveness of dapagliflozin**				
HR for all-cause death	0.83	0.71–0.97	Log-normal	^10^
HR for urgent care visit	0.43	0.20–0.90	Log-normal	^10^
HR for HF hospitalization	0.70	0.59–0.83	Log-normal	^10^
Utility of ongoing HF	0.78	0.66–0.89	Beta	^31^
Disutility of HF hospitalization	−0.100	−0.080 to −0.130	Beta	^32^
Disutility of urgent care visit	−0.036	−0.0396 to −0.0324	Beta	^18^
**Costs (2023 US$)**				
Standard treatment^d^	82.71	66.17–99.25	Gamma	BPS/MS SIGTAP/DATASUS
Dapagliflozin^e^	74.97	65.72–82.22	Gamma	BPS/MS SIGTAP/DATASUS
Urgent care visit	18.53	14.82–22.24	Gamma	SIGTAP/DATASUS
HF hospitalization	611.09	488.80–733.20	Gamma	SIH/DATASUS

All inputs are based on a quarterly cycle length. Transition probabilities for hospitalization only applied to the first cycle, and thereafter was increased following age-related trends.

Abbreviations: BPS, Brazilian Health Prices Data Bank; DATASUS, Brazilian public health system Department of Informatics; CI, confidence interval; DAPA-HF, Dapagliflozin and Prevention of Adverse Outcomes in Heart Failure; HF, heart failure; HR, hazard-ratio; SIGTAP, Brazilian Healthcare Procedures Table; SIH, Brazilian Hospital Information System; MS, Ministry of Health.

a Conversion of the DAPA-HF observed rate of 0.007 events per patient-year in the placebo group into a 3-month probability, using the formula: 1–exp(−r∗t) = 1–exp(−0.007∗1/4) = 0.002.

b Conversion of the DAPA-HF observed rate of 0.098 events per patient-year in the placebo group into a 3-month probability, using the formula: 1–exp(−r∗t) = 1–exp(−0.098∗1/4) = 0.024.

c Probability of death for the Brazilian population at age 66 (first model cycle), adjusted using a rate ratio of 4.6, as described in the Methods section.

d Includes standard HFrEF therapy drugs and additional costs related to ambulatory monitoring.

e Only drug costs.

#### Hospitalization and urgent care visit

We estimated the quarterly probability of heart failure hospitalization and urgent care visit from the DAPA-HF control arm. The observed rate of hospitalization in the trial was 9.8 per 100 patients/year, which was converted to a 3-month probability of 0.024. We calculated the higher risk of being hospitalized for heart failure as people age by using a statistical model created by McEwan et al.,^18^ which is based on the DAPA-HF trial data about patients’ characteristics and events. According to this model, the risk increases by 16% each year. The risk of death during a HF hospitalization was 9%, derived from the first Brazilian Registry of heart failure.^30^ We conservatively assumed that dapagliflozin had no effect on reducing hospital mortality. After a non-fatal hospitalization, patients in this state were at a higher risk of readmission in the next cycle. Since the DAPA-HF did not provide the readmission risk, we relied upon an observational study from Bress et al.^25^ who described a 30-day readmission rate following a heart failure hospitalization of 20% (a quarterly probability of 0.246). There were very few urgent care visits in the DAPA-HF, so we input in our model a constant 3-month risk of 0.002 after converting the trial observed rate of 0.7 per 100 patients/year. We also applied the dapagliflozin hazard-ratio for hospitalization and urgent care visit for the intervention arm, taken from the DAPA-HF trial.

### Quality of life

As there is a lack of utility data in Brazilian heart failure patients, we assumed that in our model the mean baseline utility score was 0.78 at the first cycle. This utility was estimated based on the heart failure-specific health status measured in the DAPA-HF trial, using the Kansas City Cardiomyopathy Questionnaire. The average overall score reported on the trial was 68. Since condition-specific measures like the Kansas City Cardiomyopathy Questionnaire cannot be directly used in economic evaluations, these values must be mapped to a generic health-related quality of life measure, such as the EQ-5D.^28^ The EQ-5D, commonly used in cost-utility analyses, assesses five domains (mobility, self-care, usual activities, pain/discomfort, and anxiety/depression) and provides an index ranging from 0 (death) to 1 (perfect health).^33^ We mapped the Kansas City Cardiomyopathy Questionnaire score to EQ-5D-3L utilities using a method developed by Parizo et al.^31^ based on United States time trade-off valuations. The baseline utility score was decreased by 0.174% in each quarterly cycle, to take into account the effects of aging and disease progression.^34^ For each hospitalization and readmission, there was a one-off disutility of −0.100.^32^ The estimated utility decrement associated with the incidence of an urgent care visit was −0.036.^18^

### Costs

Our model considered only direct medical costs. We extracted the acquisition costs of the standard HFrEF therapy drugs from the Brazilian Health Prices Data Bank, a national registry that discloses purchasing prices of medicines that are paid for by all levels of government.^35^ Costs identified in years other than 2023 were adjusted based on the official inflation rate estimated by the Extended National Consumer Price Index in Brazil.^36^ These costs were then calculated based on the proportion of heart failure patients using each medication on the DAPA-HF trial. The estimated cost of dapagliflozin was at US$ 299.90 yearly, almost 3-fold the standard therapy cost in the intervention arm.

There were also additional costs related to ambulatory monitoring of HFrEF patients, that were equally applied to both model arms. We assumed that all patients performed medical consultation, laboratory testing, EKG, echocardiography, and further specialized tests according to the observed frequency of a Brazilian cross-sectional study of heart failure patients.^37^ Patients with diabetes had higher background costs than patients without diabetes, due to the use of oral antidiabetic drugs, insulin, and glucose home-monitoring.

Concerning hospitalization costs, we adopted the average reimbursement of each heart failure admission according to the Brazilian Hospital Information System records for the 2023 year. The hospitalization cost was US$ 611.09, including the weighted average cost of procedures not covered in the initial admission. Urgent care visit costs were based on the Brazilian Healthcare Procedures Table, which includes the reference value of all medical procedures under SUS coverage. All costs were obtained in 2023 Brazilian currency (real, R$) and the converted into US dollars (US$) according the average official rate for the year 2023 (US$ 1: R$ 4.99).^38^ According to the Brazilian guidelines of economical evaluation, the future value of both costs and effects were discounted at an annual 5% rate.^39^ Further details on the costs of all events incorporated into the model are provided in the Supplementary material.

### Sensitivity and scenario analysis

We performed both deterministic (Tornado) and probabilistic sensitivity analyses to explore the impact of uncertainty related to epidemiological variables and costs. For the deterministic analysis, each parameter was varied one at a time across its 95% confidence interval. When there were no specified data range, we assumed ±10% variation for probability and ±20% variation for medical costs. The results of each parameter on the ICER are displayed as a tornado diagram. The bandwidth in a Tornado diagram indicates the impact of parameter variation, with the most influential parameters positioned at the top. The probabilistic sensitivity analysis was carried out using 10,000 model iteractions through a second-order Monte Carlo simulation, where input parameters values were simultaneously and randomly drawn from prespecified statistical distributions. We used beta distribution for the probability and utility parameters, gamma distribution for costs, and log-normal for HR (in the dapagliflozin arm). Probabilistic sensitivity analyses results are presented as a scatterplot and using the cost-effectiveness acceptability curve. Additionally, scenario analyses were conducted to evaluate model assumptions and test the strength of the study findings. First, the discount rate was reduced to 0% (undiscounted) and then increased to 10%. Second, the model time horizon was decreased from lifetime to 2 and 5 years. Third, the simulation model incorporated the occurrence of adverse events, including volume depletion and serious acute kidney injury, as reported in the DAPA-HF trial. The costs for these events were based on the average reimbursement for volume depletion or acute kidney injury admissions, according to the Brazilian Hospital Information System records (please check Supplementary Table S8 for further detail). Short-term quality of life penalties per incidence cycle were applied, derived from published literature (see Supplementary material for additional modelling details). Lastly, the simulated cohort was stratified by the diagnosis of diabetes at baseline. In the DAPA-HF trial, the effects of dapagliflozin on hospitalization for heart failure and death from any cause were similar among patients with and without diabetes at baseline. When compared to placebo, diabetic patients in the dapagliflozin group had a 24% reduction in the risk of hospitalization and a 22% reduction in all-cause mortality. Among non-diabetic patients, dapagliflozin reduced the risk of hospitalization by 37% and all-cause mortality by 12%.

### Validation

To evaluate and discuss the model's adequacy, the quality of the data, and the validity of the assumptions adopted, face validation was conducted through discussions with the multidisciplinary team and experts in cardiology, epidemiology, and health economics. To assess external consistency, the model and its results were compared with those of other studies addressing the same research problem. An external validation was conducted partially, by comparing the model's outcomes on mortality with real-world epidemiological data on heart failure. This approach allowed us to assess the consistency of the model's projections with observed data. However, external validation was constrained by the unavailability of accessible real-world data, thereby precluding a more comprehensive validation. The study used the best available evidence and real-world data from Brazil, particularly concerning mortality and costs.

### Role of the funding source

This study was supported by the National Institute of Science and Technology for Health Technology Assessment (Instituto de Avaliação de Tecnologias em Saúde—IATS). The funding body was not involved in the study design, data collection, data analysis, interpretation, writing of the report, or decision to publish the manuscript.

## Results

### Base case

The main results of our model are presented in Table 2. The results were estimated from a hypothetical cohort that followed 1,000 patients with HFrEF starting at age 66 and assumed a lifetime horizon. This analysis showed that the addition of dapagliflozin to standard treatment prevented 319 hospitalizations and 106 urgent visits. Furthermore, the analysis indicated that the dapagliflozin therapy resulted in 374.48 years of life saved and 366.99 QALYs gained. It was observed that the dapagliflozin strategy resulted in a total cost of US$ 3,699,287.45 for the 1,000 patients, representing an incremental cost of US$ 1,517,878.49. The cost-effectiveness analysis indicates that dapagliflozin therapy, when compared to standard therapy, has an ICER of US$ 4,764.78 per hospitalization avoided, US$ 4,053.16 per year of life saved, and US$ 4,136.08 per QALY gained.

**Table 2:** Cost-effectiveness results for the base-case analysis (per 1,000 patients).

Parameter	Dapagliflozin plus standard care	Standard care	Difference
*Clinical effectiveness*			
Hospitalizations	1,303.20	1,621.77	318.57
Urgent care visits	110.98	217.41	106.43
Life-years	5,333.01	4,958.52	374.48
QALYs	3,819.92	3,452.93	366.99
*Costs (2023 US$)*			
Medication costs	3,177,258.47	1,524,677.77	1,652,580.70
Hospitalization costs	520,313.10	653,338.78	133,025.68
Urgent care costs	1,715.88	3,392.41	1,676.53
Total costs	3,699,287.45	2,181,408.96	1,517,878.49
*Cost-effectiveness (2023 US$)*			
ICER per hospitalization avoided	–	–	4,764.78
ICER per life-year saved	–	–	4,053.16
ICER per QALY gained	–	–	4,136.08

Abbreviations: ICER, incremental cost-effectiveness ratio; QALY, quality-adjusted life year.

### Sensitivity analyses

**Fig. 2:**
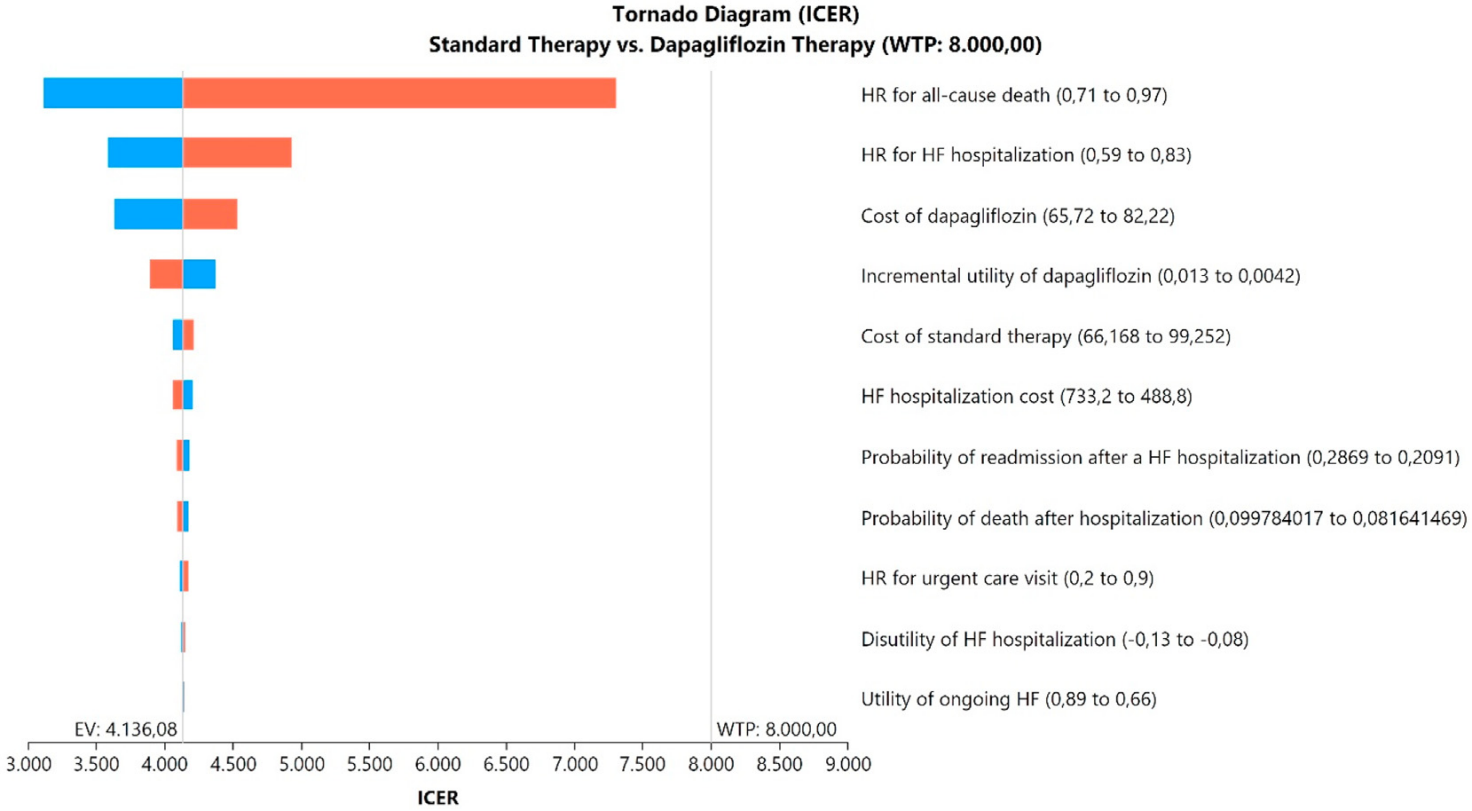
Tornado diagram (deterministic sensitivity analyses). Blue bars indicate the use of low inputs, while red bars represent high inputs. Abbreviations: ICER, incremental cost-effectiveness ratio; HF, heart failure; HR, hazard-ratio.

The epidemiological and cost variables that most impacted the ICER were presented in a tornado diagram (Fig. 2). The longer the bar, the more sensitive the ICER is to that parameter. The most influential parameters in the ICER were the HR for all-cause death and hospitalization with dapagliflozin, and its acquisition cost. Notably, the probability of death or readmission after a heart failure hospitalization had minimal impact on the result of the study. Across the 95% CI (0.71–0.97) for HR of all-cause mortality, the ICER varied from US$ 3,117–US$ 7,305. Overall, dapagliflozin was cost-effective in all the parameters varied in one-way sensitivity analysis.

**Fig. 3:**
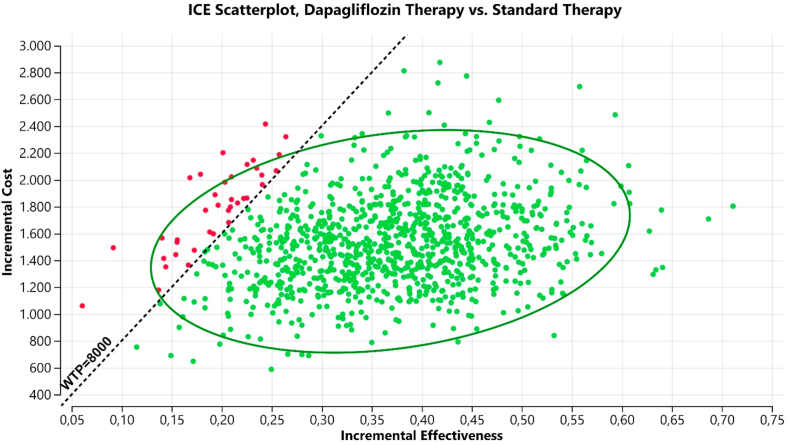
ICER scatterplot (probabilistic sensitivity analyses). Abbreviation: WTP, willingness-to-pay.

**Fig. 4:**
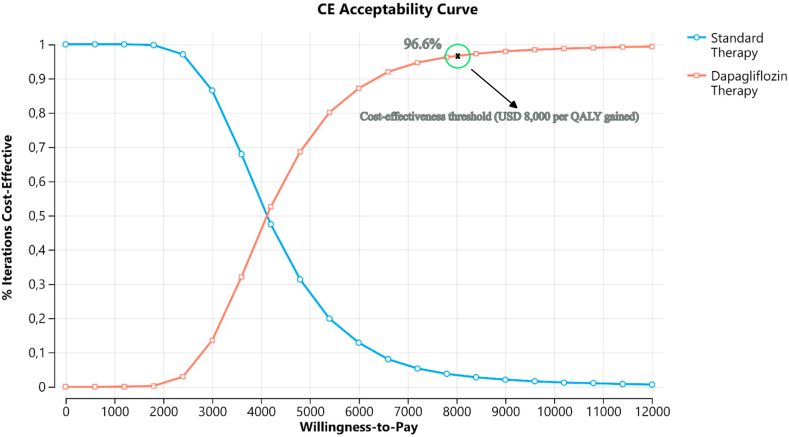
Cost-effectiveness acceptability curve.

The results of probabilistic sensitivity analyses, derived from 10,000 Monte Carlo simulations, are displayed in a scatterplot (Fig. 3). Assuming a cost-effectiveness threshold of US$ 8,000 per QALY gained, the addition of dapagliflozin to standard care was cost-effective in 96.60% of the iterations. The cost-effectiveness acceptability curve for QALY is presented in Fig. 4.

### Scenario analyses

The hypothetical scenarios tested in our model and the respective outcomes and ICERs are shown in Table 3. Varying the discount rate from 0 to 10% had little effect on the ICER. When the discount rate was set to 0%, the ICER was estimated at US$ 3,850. On the other hand, a 10% discount rate resulted in an ICER of US$ 4,433. On probabilistic sensitivity analyses, both situations had more than 99% probability of being cost-effective. Regarding the time horizon, as it became shorter the ICER gradually increased. Upon limiting the time horizon to 2 years, the ICER per QALY gained was increased to US$ 10,294. This was the only scenario where dapagliflozin would not be cost-effective. Dapagliflozin would need to be effective for at least 4.3 years to meet the willingness-to-pay threshold of US$ 8,000. The inclusion of treatment-related adverse events slightly decreased the ICER to US$ 4,037.26 per QALY gained. While dapagliflozin remained cost-effective independently of diabetes status, the ICER for diabetic patients was lower than for non-diabetic patients (US$ 3,582.57 vs US$ 4,666.73, respectively).

**Table 3:** Scenario analyses.

Category	Scenario	ICER per QALY (2023 US$)
Base-case		4,136.08
Discounting	0%	3,849.55
	10%	4,433.11
Time horizon	2 years	10,294.34
	5 years	6,098.20
Adverse events considered	Yes	4,037.26
Diabetes status	Yes	3,582.57
	No	4,666.73

Abbreviations: ICER, incremental cost-effectiveness ratio; QALY, quality-adjusted life year.

## Discussion

The base-case analysis for this simulation model of HFrEF in Brazil showed that the adjunct use of dapagliflozin to standard care is highly cost-effective compared to standard care alone. The yielded ICER of US$ 4,136.08 per QALY gained was approximately half of the Brazilian willingness-to-pay threshold of US$ 8,000 per QALY gained. The observation that dapagliflozin was cost-effective in 96.60% of 10,000 simulations implies that our findings hold steady across a broad range of key model parameter estimates. The ICERs for hospitalization avoided and years of life saved provide supplementary data that corroborate the cost-effectiveness of the dapagliflozin therapy. Moreover, the analysis indicates that dapagliflozin therapy should result in a reduction in the number of hospitalizations and emergency room visits over the projected time horizon, accompanied by an increase in the number of QALYs. Specifically, the study estimates that for every 1,000 patients, approximately 319 hospitalizations and 106 emergency room visits would be avoided, and 367 QALYs would be gained.

The outcomes were particularly sensitive to the mortality impact and cost of dapagliflozin. Hence, it is of paramount importance to conduct a more precise evaluation of dapagliflozin's effect on mortality. According to the 95% CI used in the DAPA-HF trial, the all-cause mortality spanned from a reduction of 3%–29%. However, even when using on the model this lower limit of a 3% mortality reduction, the cost-effectiveness of dapagliflozin still remains. Although in the DAPA-HF trial the HR of dapagliflozin for cardiovascular death and death for any cause were similar (0.82 and 0.83, respectively), there were fewer non-cardiovascular deaths in the dapagliflozin arm. This suggests the effect of other beneficial mechanisms of SGLT2 inhibition, thus affecting the economic benefits of dapagliflozin.

Heart failure and diabetes have a well-established association, broadening the risk of adverse outcomes. Individuals with diabetes have a two to fourfold risk of developing heart failure, while in instance the prevalence of diabetes in heart failure patients is higher than in the general population.^40^ Diabetes triggers processes that accelerate atherosclerosis and coronary artery disease, a major cause of HFrEF worldwide and in Brazil.^7^ Moreover, the metabolic disturbances associated with diabetes promote cardiac stiffness and diastolic dysfunction, ultimately precipitating HF due to diabetic cardiomyopathy.^41^ A subgroup analysis of the DAPA-HF indicated that dapagliflozin lowers the risk of cardiovascular deaths by 15% in patients without diabetes and 21% in patients with diabetes, providing evidence that the benefits of SGLT2 inhibition are not limited to people with diabetes.^42^ In our model, a scenario analysis demonstrated that dapagliflozin had similar cost-effectiveness among patients with and without diabetes.

The DAPA-HF trial showed no significant difference in safety outcomes between the control and intervention groups. Our results remained consistent when treating volume depletion and serious kidney injury as transitory events, with a modest ICER reduction to US$ 4,037.26. This reflects the higher incidence of serious renal adverse events in the standard of care control group (2.7%) compared to the dapagliflozin group (1.6%) observed in the trial. The beneficial effects of dapagliflozin on renal function were further investigated and confirmed in the DAPA-CKD trial.^43^

Previous cost-effective analyses have already demonstrated that dapagliflozin is cost-effective among patients with HFrEF. There are, however, notable differences in the estimates of long-term effectiveness of dapagliflozin between studies from different countries regardless of income level. Our estimate of 0.37 QALYs per person gained are closer to the gain of 0.38 QALYs in the Chinese model^16^ and to the gain of 0.37 QALYs in the Egyptian model,^44^ but somewhat different from the gain of 0.60 QALYs estimated for the Thai population.^45^ Prior analyses performed from the perspective of health systems from high-income countries also evidenced diverse estimates for the effectiveness of dapagliflozin. Isaza et al.^14^ estimated a gain of 0.63 QALYs in the United States, while Savira et al.^15^ estimated a gain of 0.29 QALYs in Australia. Despite using the same source for clinical data on the effectiveness of dapagliflozin, we attribute these differences to the considerable variation in model assumptions across distinct cost-effectiveness analysis. Of note, diverse cycle lengths, time horizons, and quality of life estimates can lead to different levels of accumulated QALYs.

The ICER also varied substantially in previous studies. The ICER of US$ 4,136 per QALY gained estimated in our study is markedly lower than the ICER of US$ 12,305 in Taiwan,^17^ being closer to the ICER of US$ 3,638 in the Philippines,^46^ and higher than the ICER of US$ 1,991 estimated in Thailand.^45^ Although all these studies found that dapagliflozin was cost-effective, clear gasps remain in the level of cost-effectiveness analysis evidence between higher-income and lower-income countries.^47^ Even though 80% of the global cardiovascular disease deaths occurred in low- and middle-income countries between 1990 and 2019,^48^ available epidemiological and economic evidence on cardiovascular disease in higher-income countries far exceeds that found in lower-income countries. This evidence, however, may not be directly transferable or generalizable to low- and middle-income countries.^49^ A systematic review of cost-effectiveness analysis of dapagliflozin in the management of HFrEF emphasized the need for more economic evaluations in developing countries.^50^

Latin America and the Caribbean comprise a heterogeneous group of countries. Most of the epidemiological and economic studies on heart failure patients have been conducted in Brazil and Argentina,^7^ which have relatively high per capita incomes for the area. Using an alternative approach to estimating cost-effectiveness thresholds developed by Pichon-Riviere et al.,^51^ our results would not be considered cost-effective for lower-middle income countries in the region. The estimated ICER of US$ 4,136 per QALY gained is above the threshold of US$ 1,889 in Bolivia and US$ 1,603 in Honduras.

Costs concerns are more critical in resource-constrained health systems in low- and middle-income countries of Latin America and the Caribbean. It is worth noting that treatments for HFrEF could significantly impact healthcare budgets due to the large number of patients eligible for lifelong therapy.^14^ However, for SGLT-2 inhibitors such as dapagliflozin the projected increase in costs may be overestimated, as these medications are already routinely prescribed for type 2 diabetes mellitus.^50^ A formal Budget Impact Analysis would be required to assess the financial consequences of adopting dapagliflozin within the Brazilian healthcare budget.

The National Commission for the Incorporation of Health Technologies (CONITEC) in Brazil, established in 2011, advises the Ministry of Health on incorporating new medicines into the SUS. Although dapagliflozin was recommended by CONITEC as cost-effective in 2020,^52^ it remains largely inaccessible to many patients in need. Implementation of new technologies is typically slow, primarily due to challenges with decentralized operations.^53^^,^^54^ Local pharmaceutical care management is generally limited to operational aspects, highlighting an urgent need for investments in sustainability and accessibility. Particularly, there is a need for clear and coordinated actions, such as increased social oversight, improved infrastructure in peripheral pharmacies, and better integration with other levels of healthcare.^55^ Failure to overcome barriers limiting access to pharmaceutical care may compromise adherence, as most of the Brazilian population cannot afford newer medications like dapagliflozin. Besides leading to poor health outcomes, nonadherence also drives up health care expenses, which are unsustainable for the health systems in low- and middle-income countries.^56^ Conversely, enhanced adherence can result in substantial decrease in healthcare costs. Improving adherence requires a continuous, dynamic process involving patient-tailored interventions and a multidisciplinary approach. Strategies such as individual counseling, medication education, family support, and nurse practitioner-based services have demonstrated effectiveness in promoting long-term medication use.^57^

This study has some limitations. First, most of the transition probabilities and effects were derived from a single trial with a relatively short follow-up of 18 months. Although the trial was large and consistent with the literature on SGLT2 inhibitors use in HFrEF patients, our findings are primarily applicable to populations with similar characteristics to the cohort studied and may be limited to populations with different profiles. Additionally, the cost-effectiveness of dapagliflozin may be overestimated if its long-term effectiveness diminishes over an extended time horizon. We predicted long-term survival outcomes in a parametric way, combining trial data and Brazilian life tables. The uncertainty introduced by this model was assessed in the sensitivity analyses, and the cost-effectiveness kept its robustness. We assumed in our study a 100% adherence to dapagliflozin, a highly unlikely situation in the real world. Although dapagliflozin adherence was not reported in the DAPA-HF trial, it did report a very similar and low probability of discontinuation for any adverse event between treatment groups. Although non-adherence may affect the effectiveness of the therapeutic strategies under evaluation, the study results would not be significantly affected, since both benefits and costs would be proportionally impacted across strategies, thus mitigating the effects on the ICERs. Furthermore, we only investigated heterogeneity based on diabetes status at baseline, excluding factors such as symptom severity, age, or sex in our model. However, the DAPA-HF trial demonstrated that dapagliflozin was effective across all prespecified subgroups, including these variables. It is important to note that the study focused its analysis on the general population of HFrEF patients aged 66 and over, using aggregate estimates for risks, benefits, and costs. Finally, we applied in our model utility data from the DAPA-HF trial due to the scarcity of local data. The mean baseline utility score for heart failure patients was, nevertheless, remarkably similar to the general Brazilian population norms for EQ-5D-3L, stratified by age.^58^

In conclusion, this economic evaluation study suggested that from the Brazilian public healthcare perspective, adding dapagliflozin to standard therapy in HFrEF patients is likely to be cost-effective compared to standard therapy alone, regardless of diabetes status. Further evaluation is warranted to investigate the long-term and real-world effects of dapagliflozin on the Brazilian population. Furthermore, a budget impact analysis would clarify if dapagliflozin is also affordable for the Brazilian public health system.

## Contributors

MCXN, AAA and HMP conceived and designed the initial proposal. Literature search and data collection were performed by MCXN. HMP and MCXN formally analyzed the data. IRZ and HMP elaborated and reviewed the methodology. MCXN wrote the initial draft that was edited and revised by AAA, IRZ and HMP. All authors contributed to the interpretation of the results, critical review, approval of the final text and were responsible for the decision to submit the manuscript.

## Data sharing statement

All data generated and analyzed in this study are openly accessible through the sources listed in Table 1. A more detailed dataset used or analyzed during the current study is available from the corresponding author upon reasonable request.

## Editorial disclaimer

The translation of the Summary was submitted by the authors, and we reproduce it as supplied. It has not been peer reviewed. Our editorial processes have only been applied to the original version in English, which should serve as a reference for this manuscript.

## Declaration of interests

None declared.

## Appendix A Supplementary data

Supplementary Material

Abstract (portuguese)
