# Supplementary material for: Dapagliflozin for the treatment of heart failure with reduced ejection fraction in Brazil: a cost-effectiveness analysis
Source: Lancet Reg Health Am. 2024 Dec 28;42:100968. doi: 10.1016/j.lana.2024.100968 (PMC11742827; doi:10.1016/j.lana.2024.100968)
Supplement: Supplementary Material [file mmc1.docx]

**Dapagliflozin for the Treatment of Heart Failure with Reduced Ejection Fraction in Brazil: A Cost-Effectiveness Analysis**

**Supplementary Material**

**Summary**

[**1.** **Supplementary Methods** 1](#_Toc184303522)

[1.2. Urgent heart failure visits 1](#_Toc184303523)

[1.2. Heart failure hospitalization 2](#_Toc184303524)

[1.3. Background healthcare 3](#_Toc184303525)

[**2.** **Supplementary Tables** 4](#_Toc184303526)

[2.1. Supplementary Table 1. All-cause mortality probability by cohort and age (annual %) 4](#_Toc184303527)

[2.2. Supplementary Table 2. Standard therapy costs 5](#_Toc184303528)

[2.3. Supplementary Table 3. Dapagliflozin cost 6](#_Toc184303529)

[2.4. Supplementary Table 4. Background healthcare costs 7](#_Toc184303530)

[2.5. Supplementary Table 5. Additional costs for diabetic patients 8](#_Toc184303531)

[2.6. Supplementary Table 6. Urgent visit costs 9](#_Toc184303532)

[2.7. Supplementary Table 7. Admission costs 10](#_Toc184303533)

[2.8. Supplementary Table 8. Parameters for adverse events (scenario analysis) 11](#_Toc184303534)

[**CHEERS Checklist (2022)** 12](#_Toc184303535)

[**References** 14](#_Toc184303536)

# **Supplementary Methods**

## **Urgent heart failure visits**

An urgent care visit was defined in the DAPA-HF trial as an urgent, unscheduled office/practice or emergency department visit for a primary diagnosis of heart failure, but not meeting the criteria for a heart failure hospitalization. In this visit, the patient receives initiation or intensification of treatment specifically for heart failure. We assumed that every urgent heart failure visit included a set of laboratory studies (complete blood count, blood chemistry with electrolytes, kidney function tests, liver function tests, urinary analysis, and brain natriuretic peptide), an electrocardiogram and a chest X-ray. We also assumed that 20% of the patients undergo transthoracic echocardiography during that visit. Based on expert opinion, we assumed that every visit required an intravenous infusion of furosemide (40mg). All the costs were obtained from SIGTAP (SUS Procedures Table Management System), accessed in December 2023. Supplementary table 5 shows detailed costs of charges included in the calculation of the urgent heart failure visit cost.

## **1.2. Heart failure hospitalization**

According to the DAPA-HF Study Protocol, a heart failure hospitalization was defined as an event where the patient was admitted to the hospital with a primary diagnosis of heart failure due to worsening symptoms, and the patient's length-of-stay in hospital extends for at least 24 hours. The cost of heart failure hospitalization was estimated from the average reimbursement of each HF admission according to the Brazilian Hospital Information System (SIH/SUS) for the 2023 year, using the International Code of Diseases (ICD) I50. Based on the first Brazilian Registry of heart failure ^1^, we assumed that 1·2% of admitted heart failure patients would have an indication for a pacemaker and 1·7% would have an indication for an implantable cardioverter-defibrillators (ICD). These additional costs were obtained from SIGTAP (SUS Procedures Table Management System), accessed in December 2023. Supplementary table 6 shows detailed costs of charges included in the calculation of the hospitalization cost.

## **Background healthcare**

The optimal frequency of medical consultations for patient with heart failure and reduced ejection fraction is not clearly defined in the literature. Based on expert opinion and our own institutional experience, we assumed that every patient had four scheduled visits during a year. In every visit, the patient had a laboratory workup as directed by the 2022 AHA/ACC/HFSA Guideline for the Management of Heart Failure.^2^ This laboratory evaluation included complete blood count, urinalysis, serum electrolytes (including sodium, potassium, calcium, and magnesium), blood urea nitrogen, serum creatinine, glucose, fasting lipid profile, liver function tests, iron studies (serum iron, ferritin, transferrin saturation), and thyroid-stimulating hormone level. We assumed that the patients would undergo a chest X-ray twice and a transthoracic echocardiography once a year. The frequency of additional exams (treadmill stress test, cardiac stress scintigraphy and heart catheterization) was obtained from a Brazilian cross-sectional study of heart failure patients.^3^ All the costs were obtained from SIGTAP (SUS Procedures Table Management System), accessed in December 2023. Supplementary table 3 depicts the detailed costs for background health care.

# **Supplementary Tables**

## **2.1. Supplementary Table 1. All-cause mortality probability by cohort and age (annual %)**

| **Age** | **Base-case** | **Diabetes** | **No diabetes** |
| --- | --- | --- | --- |
| 66 | 0·080 | 0·117 | 0·066 |
| 67 | 0·087 | 0·128 | 0·072 |
| 68 | 0·094 | 0·138 | 0·078 |
| 69 | 0·101 | 0·149 | 0·084 |
| 70 | 0·109 | 0·160 | 0·091 |
| 71 | 0·118 | 0·173 | 0·098 |
| 72 | 0·128 | 0·188 | 0·106 |
| 73 | 0·140 | 0·205 | 0·116 |
| 74 | 0·153 | 0·225 | 0·127 |
| 75 | 0·169 | 0·248 | 0·140 |
| 76 | 0·186 | 0·272 | 0·154 |
| 77 | 0·204 | 0·299 | 0·169 |
| 78 | 0·222 | 0·326 | 0·184 |
| 79 | 0·242 | 0·355 | 0·201 |
| 80 | 0·263 | 0·386 | 0·218 |
| 81 | 0·288 | 0·422 | 0·239 |
| 82 | 0·317 | 0·465 | 0·263 |
| 83 | 0·352 | 0·516 | 0·292 |
| 84 | 0·392 | 0·576 | 0·326 |
| 85 | 0·437 | 0·641 | 0·363 |
| 86 | 0·483 | 0·708 | 0·401 |
| 87 | 0·527 | 0·773 | 0·437 |
| 88 | 0·567 | 0·832 | 0·471 |
| 89 | 0·603 | 0·885 | 0·501 |
| 100 | 1 | 1 | 1 |

## **Supplementary Table 2. Standard therapy costs**

| **Drug** | **Dose ^a^** | **Unitary cost (R$)** | **Use ^b^ (%)** | **Annual cost (R$)** | **Source ^c^** |
| --- | --- | --- | --- | --- | --- |
| Enalapril | 20mg twice daily | 0·05 | 56 | 20·48 | BPS/MS |
| Losartan | 100mg once daily | 0·07 | 27 | 13·64 | BPS/MS |
| Sacubitril/valsartan | 97/103mg twice daily | 3·35 | 11 | 266·56 | BPS/MS |
| Spironolactone | 25mg once daily | 0·14 | 71 | 36·08 | BPS/MS |
| Carvedilol | 25mg twice daily | 0·17 | 96 | 119·38 | BPS/MS |
| Digoxin | 0.125mg once daily | 0·13 | 19 | 4·41 | BPS/MS |
| Furosemide | 40mg twice daily | 0·06 | 93 | 40·52 | BPS/MS |

^a^ Target dose according to the 2022 AHA/ACC/HFSA Guideline for the Management of Heart Failure, except for furosemide and digoxin whose doses are the usual for HF patients

^b^ Proportion of patients (%) in the DAPA-HF trial using the drug

**^c^** All the medication costs were obtained from the Brazilian Health Prices Data Bank (BPS), accessed in December 2023.

## **Supplementary Table 3. Dapagliflozin cost**

| **Drug** | **Dose** | **Unitary cost (R$)** | **Use (%)** | **Annual cost (R$)** | **Source ^a^** |
| --- | --- | --- | --- | --- | --- |
| Dapagliflozin | 10mg once daily | 4·10 | 100 | 1,496·50 | BPS/MS |

^a^ Brazilian Health Prices Data Bank (BPS), accessed in December 2023.

## **Supplementary Table 4. Background healthcare costs**

| **Procedure** | **Use (%)** | **Annual quantity** | **Unitary cost (R$)** | **Annual cost (R$)** |
| --- | --- | --- | --- | --- |
| Cardiology visit | 100 | 4 | 10·00 | 40·00 |
| Electrocardiogram (EKG) | 100 | 4 | 5·15 | 20·60 |
| Complete blood count | 100 | 4 | 4·11 | 16·44 |
| Aspartate transaminase (AST) | 100 | 4 | 2·01 | 8·04 |
| Alanine transaminase (ALT) | 100 | 4 | 2·01 | 8·04 |
| Blood Urea Nitrogen | 100 | 4 | 1·85 | 7·40 |
| Serum Creatinine | 100 | 4 | 1·85 | 7·40 |
| Serum Sodium | 100 | 4 | 1·85 | 7·40 |
| Serum Potassium | 100 | 4 | 1·85 | 7·40 |
| Serum Calcium | 100 | 4 | 1·85 | 7·40 |
| Serum Magnesium | 100 | 4 | 2·01 | 8·04 |
| Glucose | 100 | 4 | 1·85 | 7·40 |
| HbA1C | 100 | 4 | 7·86 | 31·44 |
| Total cholesterol | 100 | 4 | 1·85 | 7·40 |
| HDL cholesterol | 100 | 4 | 3·51 | 14·04 |
| LDL cholesterol | 100 | 4 | 3·51 | 14·04 |
| Triglycerides | 100 | 4 | 3·51 | 14·04 |
| Thyroid-stimulating hormone | 100 | 4 | 8·96 | 35·84 |
| Serum iron | 100 | 4 | 3·51 | 14·04 |
| Ferritin | 100 | 4 | 15·59 | 62·36 |
| Transferrin saturation | 100 | 4 | 2·01 | 8·04 |
| Urinalysis | 100 | 4 | 3·70 | 14·80 |
| Brain Natriuretic Peptide | 100 | 1 | 27·00 | 27·00 |
| Transthoracic echocardiogram | 100 | 1 | 39·94 | 39·94 |
| Chest X-ray | 100 | 2 | 9·50 | 19·00 |
| Treadmill stress test | 8 | 1 | 30·00 | 2·40 |
| Cardiac stress scintigraphy | 15 | 1 | 408·52 | 61·28 |
| Heart catheterization | 14 | 1 | 614·72 | 86·06 |

Source: SIGTAP (SUS Procedures Table Management System), accessed in December 2023.

## **Supplementary Table 5. Additional costs for diabetic patients**

| **Drug / procedure** | **Dose** | **Unitary cost (R$)** | **Use (%)** | **Annual cost (R$)** |
| --- | --- | --- | --- | --- |
| Metformine | 850mg 3 times a day | 0·07 | 52 | 39·63 |
| Glicazide | 120mg once daily | 0·27 | 21 | 41·79 |
| Linagliptine | 5mg once daily | 6·16 | 15 | 339·51 |
| Semaglutide | 0,5mg weekly | 622·71 | 1 | 37·36 |
| NPH insulin | 40 UI / day | 19·34 | 27 | 759·56 |
| Syringes | 1 syringe / vial | 0·39 | 27 | 15·32 |
| Needles | 1 needle / application | 0·10 | 27 | 19·64 |
| Glucose monitor | 1 unity / 5y | 37·46 | 100 | 7·49 |
| Stripes | 120 unitys / year | 0·31 | 100 | 37·20 |
| Oftalmology visit | N/A | 10·00 | 100 | 10·00 |
| Microalbuminure | N/A | 8·12 | 100 | 8·12 |

Source: Brazilian Health Prices Data Bank (BPS), accessed in December 2023.

## **Supplementary Table 6. Urgent visit costs**

| **Procedure** | **Use (%)** | **Quantity** | **Unitary cost (R$)** | **Total cost (R$)** |
| --- | --- | --- | --- | --- |
| Urgent visit | 100 | 1 | 10·00 | 10·00 |
| Electrocardiogram (EKG) | 100 | 1 | 5·15 | 5,15 |
| Complete blood count | 100 | 1 | 4·11 | 4·11 |
| Aspartate transaminase (AST) | 100 | 1 | 2·01 | 2·01 |
| Alanine transaminase (ALT) | 100 | 1 | 2·01 | 2·01 |
| Blood Urea Nitrogen | 100 | 1 | 1·85 | 1·85 |
| Serum Creatinine | 100 | 1 | 1·85 | 1·85 |
| Serum Sodium | 100 | 1 | 1·85 | 1·85 |
| Serum Potassium | 100 | 1 | 1·85 | 1·85 |
| Serum Calcium | 100 | 1 | 1·85 | 1·85 |
| Serum Magnesium | 100 | 1 | 2·01 | 2·01 |
| Glucose | 100 | 1 | 1·85 | 1·85 |
| Brain Natriuretic Peptide | 100 | 1 | 27,00 | 27·00 |
| Urinalysis | 100 | 1 | 3·70 | 3·70 |
| Chest X-ray | 100 | 1 | 9·50 | 9·50 |
| Transthoracic echocardiogram | 20 | 1 | 67·86 | 13·57 |
| Furosemida IV 20mg | 100 | 2 | 1·16 | 2·32 |

Source: SIGTAP (SUS Procedures Table Management System) and Brazilian Health Prices Data Bank (BPS), accessed in December 2023.

## **Supplementary Table 7. Admission costs**

| **Procedure** | **Use (%)** | **Quantity** | **Unitary cost (R$)** | **Total cost (R$)** | **Source** |
| --- | --- | --- | --- | --- | --- |
| Hospital admission | 100% | 1 | 2,330·03 | 2,330·03 | SIH/DATASUS 2023 |
| Pacemaker implantation | 1% | 1 | 6,450·89 | 77·41 | SIGTAP 12/23 |
| Implantable cardioverter-defibrillator | 2% | 1 | 38,118·28 | 648·01 | SIGTAP 12/23 |

Abbreviations: SIH/DATASUS, Brazilian Hospital Information System; SIGTAP, SUS Procedures Table Management System.

## **Supplementary Table 8. Parameters for adverse events (scenario analysis)**

| **Event** | **Incidence % (control)** | **Incidence % (dapagliflozin)** | **Cost (2023 US$)** | **Disutility** |
| --- | --- | --- | --- | --- |
| Volume depletion | 1·7^a^ | 1·2^b^ | 768·63 | -0·051 |
| Acute kidney injury | 2·7^c^ | 1·6 | 55·30 | -0·076 |

# **CHEERS Checklist (2022)**

|  | **Item** | **Guidance for Reporting** | **Reported in section** |
| --- | --- | --- | --- |
| **TITLE** | | |  |
| Title | 1 | Identify the study as an economic evaluation and specify the interventions being compared. | Page 1 |
| **ABSTRACT** | | |  |
| Abstract | 2 | Provide a structured summary that highlights context, key methods, results and alternative analyses. | Page 2 |
| **INTRODUCTION** | | |  |
| Background and objectives | 3 | Give the context for the study, the study question and its practical relevance for decision making in policy or practice. | Page 3-4 |
| **METHODS** | | |  |
| Health economic  analysis plan | 4 | Indicate whether a health economic analysis plan was developed and  where available. | Page 5 |
| Study population | 5 | Describe characteristics of the study population (such as age range, demographics, socioeconomic, or clinical characteristics). | Page 6 |
| Setting and location | 6 | Provide relevant contextual information that may influence findings. | Page 5 |
| Comparators | 7 | Describe the interventions or strategies being compared and why chosen. | Page 5 |
| Perspective | 8 | State the perspective(s) adopted by the study and why chosen. | Page 5 |
| Time horizon | 9 | State the time horizon for the study and why appropriate. | Page 5 |
| Discount rate | 10 | Report the discount rate(s) and reason chosen. | Page 9 |
| Selection of outcomes | 11 | Describe what outcomes were used as the measure(s) of benefit(s) and harm(s). | Page 5 |
| Measurement of outcomes | 12 | Describe how outcomes used to capture benefit(s) and harm(s) were measured. | Page 7-8 |
| Valuation of outcomes | 13 | Describe the population and methods used to measure and value outcomes. | Page 6-8 |
| Measurement and valuation of resources  and costs | 14 | Describe how costs were valued. | Page 8-9 |
| Currency, price date, and conversion | 15 | Report the dates of the estimated resource quantities and unit costs, plus the currency and year of conversion. | Page 9 |
| Rationale and  description of model | 16 | If modelling is used, describe in detail and why used. Report if the model is publicly available and where it can be accessed. | Page 6 (Figure 1) |
| Analytics and assumptions | 17 | Describe any methods for analysing or statistically transforming data, any extrapolation methods, and approaches for validating any model used. | Page 10 |
| Characterizing heterogeneity | 18 | Describe any methods used for estimating how the results of the study vary for sub-groups. | Page 10 (Suppl.Table 1) |
| Characterizing  distributional effects | 19 | Describe how impacts are distributed across different individuals  or adjustments made to reflect priority populations. | NA |
| Characterizing uncertainty | 20 | Describe methods to characterize any sources of uncertainty in the analysis. | Page 9-10 |
| Approach to engagement with patients and others affected by the study | 21 | Describe any approaches to engage patients or service recipients, the general public, communities, or stakeholders (e.g., clinicians or payers) in the design of the study. | NA |
| **RESULTS** | | |  |
| Study parameters | 22 | Report all analytic inputs (e.g., values, ranges, references) including uncertainty or distributional assumptions. | Page 11 |
| Summary of main results | 23 | Report the mean values for the main categories of costs and outcomes of interest and summarise them in the most appropriate overall measure. | Page 11 (Table 2) |
| Effect of uncertainty | 24 | Describe how uncertainty about analytic judgments, inputs, or projections affect findings. Report the effect of choice of discount rate and time horizon, if applicable. | Page 11-12 (Table 3, Figure 2, Figure 3 and Figure 4) |
| Effect of engagement with patients and others affected by the study | 25 | Report on any difference patient/service recipient, general public, community, or stakeholder involvement made to the approach or findings of the study | NA |
| **DISCUSSION** | | |  |
| Study findings, limitations, generalizability, and current knowledge | 26 | Report key findings, limitations, ethical or equity considerations not captured, and how these could impact patients, policy, or practice. | Page 12-16 |
| **OTHER RELEVANT INFORMATION** | | | |
| Source of funding | 27 | Describe how the study was funded and any role of the funder in the identification, design, conduct, and reporting of the analysis | Page 10 |
| Conflicts of interest | 28 | Report authors conflicts of interest according to journal or  International Committee of Medical Journal Editors requirements. | Page 16 |

Husereau D, Drummond M, Augustovski F, de Bekker-Grob E, Briggs AH, Carswell C, Caulley L, Chaiyakunapruk N, Greenberg D, Loder E, Mauskopf J, Mullins CD, Petrou S, Pwu RF, Staniszewska S; CHEERS 2022 ISPOR Good Research Practices Task Force. Consolidated Health Economic Evaluation Reporting Standards 2022 (CHEERS 2022) Statement: Updated Reporting Guidance for Health Economic Evaluations. BMJ. 2022;376:e067975.

The checklist is Open Access distributed in accordance with the terms of the Creative Commons Attribution (CC BY 4.0) license, which permits others to distribute, remix, adapt and build upon this work, for commercial use, provided the original work is properly cited. See: http://creativecommons.org/licenses/by/4.0/.

# **References**

1. Albuquerque DC, Neto JDS, Bacal F, de *et al.* I Brazilian Registry of Heart Failure - Clinical Aspects, Care Quality and Hospitalization Outcomes. *Arq Bras Cardiol* 2015;104(**6**): 433-442.
2. Heidenreich PA, Bozkurt B, Aguilar D, *et al.* 2022 AHA/ACC/HFSA Guideline for the Management of Heart Failure: A Report of the American College of Cardiology/American Heart Association Joint Committee on Clinical Practice Guidelines. *J Am Coll Cardiol* 2022;**79**:e263–e421.
3. Araujo DV, Tavares LR, Verissimo R, Ferraz MB, Mesquita ET*.* Custo Da Insuficiência Cardíaca No Sistema Único de Saúde Cost of Heart Failure in the Unified Health System. *Arquivos Brasileiros de Cardiologia* 2005;**84**:422–427.
