# Supplementary material for: Dapagliflozin for the treatment of heart failure with reduced ejection fraction in Brazil: a cost-effectiveness analysis
Source: Lancet Reg Health Am. 2024 Dec 28;42:100968. doi: 10.1016/j.lana.2024.100968 (PMC11742827; doi:10.1016/j.lana.2024.100968)
Supplement: Abstract (portuguese) [file mmc2.docx]

**Editorial disclaimer**

The translation of the Summary was submitted by the authors, and we reproduce it as supplied. It has not been peer reviewed. Our editorial processes have only been applied to the original version in English, which should serve as a reference for this manuscript.

**RESUMO**

**Contexto**

A insuficiência cardíaca (IC), uma síndrome clínica complexa com alta morbidade e mortalidade, é um grave problema de saúde pública com impacto econômico cada vez maior. Recentemente, uma nova classe de agentes antidiabéticos - os inibidores do cotransportador de sódio-glicose 2 (SGLT2i) - foi associada a uma redução significativa na mortalidade e hospitalização em pacientes com IC com fração de ejeção reduzida (ICFEr) quando adicionada ao tratamento farmacológico padrão. Considerando a escassez de dados sobre a sua relação de custo-efetividade, o presente estudo tem como objetivo estimar a relação de custo-efetividade incremental do tratamento adicional com dapagliflozina para ICFEr sob a perspectiva do sistema público de saúde brasileiro (SUS).

**Métodos**

Construímos um modelo de Markov para avaliar os resultados clínicos e os custos de 1.000 indivíduos hipotéticos com ICFEr, em um horizonte de vida inteira. Os parâmetros do modelo foram baseados no estudo *Dapagliflozin and Prevention of Adverse Outcomes in Heart Failure* (DAPA-HF) e em dados locais. O principal desfecho foi a razão de custo-efetividade incremental (RCEI) por ano de vida ganho ajustado pela qualidade (QALY). Análises de sensibilidade determinísticas e probabilísticas, bem como análises de cenário, foram realizadas.

**Resultados**

A adição de dapagliflozina ao tratamento padrão em 1.000 pacientes com ICFEr produziu uma média de 366,99 QALYs adicionais a um custo incremental de US$ 1.517.878,49, resultando em uma RCEI de US$ 4.136 por QALY ganho. Esta é uma estratégia custo-efetiva, considerando o limiar brasileiro oficial de custo-efetividade (US$ 8.000/QALY). Em análises de sensibilidade probabilística, 96,6% das simulações foram custo-efetivas. Nas análises de cenário, os resultados foram semelhantes para indivíduos com e sem diabetes.

**Interpretação**

A adição da dapagliflozina à terapia padrão para ICFEr no Brasil é provavelmente custo-efetiva.

**Financiamento**

Este estudo foi apoiado pelo Instituto Nacional de Ciência e Tecnologia para Avaliação de Tecnologias em Saúde (IATS).

**Palavras-chave:** insuficiência cardíaca, dapagliflozina, custo-efetividade, QALY, Brasil.
